# Assessment of Arrow-of-Time Metrics for the Characterization of Underwater Explosions

**DOI:** 10.3390/s21175952

**Published:** 2021-09-04

**Authors:** Ramón Miralles, Guillermo Lara, Alicia Carrión, Manuel Bou-Cabo

**Affiliations:** 1 Institute of Telecommunications and Multimedia Applications (iTEAM), Universitat Politècnica de València, 46022 Valencia, Spain; 2 Spanish Institute of Oceanography, San Pedro del Pinatar, 30740 Murcia, Spain; guillermo.lara@ieo.es (G.L.); manuel.bou@ieo.es (M.B.-C.); 3 Instituto de Instrumentación para Imagen Molecular (i3M), Consejo Superior de Investigaciones Científicas-Universitat Politècnica de València (UPV-CSIC), 46022 Valencia, Spain; alcarga4@i3m.upv.es

**Keywords:** passive acoustic monitoring, surveillance, underwater explosions, arrow of time, impulsive event detection, anthropogenic noise characterization

## Abstract

Anthropogenic impulsive sound sources with high intensity are a threat to marine life and it is crucial to keep them under control to preserve the biodiversity of marine ecosystems. Underwater explosions are one of the representatives of these impulsive sound sources, and existing detection techniques are generally based on monitoring the pressure level as well as some frequency-related features. In this paper, we propose a complementary approach to the underwater explosion detection problem through assessing the arrow of time. The arrow of time of the pressure waves coming from underwater explosions conveys information about the complex characteristics of the nonlinear physical processes taking place as a consequence of the explosion to some extent. We present a thorough review of the characterization of arrows of time in time-series, and then provide specific details regarding their applications in passive acoustic monitoring. Visibility graph-based metrics, specifically the direct horizontal visibility graph of the instantaneous phase, have the best performance when assessing the arrow of time in real explosions compared to similar acoustic events of different kinds. The proposed technique has been validated in both simulations and real underwater explosions.

## 1. Introduction

Underwater explosions (UNDEX) are one of the loudest sounds that can be heard in the ocean and can disrupt everything from tiny plankton to blue whales [1,2]. These sounds are a direct hazard to marine life and the environment, not only causing confusion but also shock waves that can produce permanent damage in the internal organs of many species [3]. For this reason, UNDEX are strongly regulated. In the European Union (EU), for instance, the Marine Strategy Framework Directive (MSFD) was adopted in 2008 and revised in 2017 in the descriptor D11C1 to regulate the spatial distribution, temporal extent, and levels of anthropogenic impulsive sound sources (which includes UNDEX) [4]. This regulation defines maximum threshold levels for the different kinds of impulsive sound, above which the sound must be reported to the corresponding authorities and included in a national register. Mitigation activities must also be implemented if needed. Examples of controlled uses of UNDEX include deepening of harbors and channels, excavation of trenches for installing oil and gas pipelines and communication cables, demolition work or removal of offshore structures, and excavation for foundations (civil engineering) [5].

Even though the use of UNDEX for fishing (also known as blast fishing) is illegal and has almost been eradicated, it is still seen in some areas in the world [6]. This is a highly destructive method that destroys habitats (coral reefs in most cases), resulting in a drastic reduction in catches and affecting food security.

All of these activities produce characteristic acoustic events that can be automatically detected [7,8] and employed to devise law enforcement tools and control mechanisms. It would be desirable to develop UNDEX detection procedures that do not just rely on the reported data. They should also rely on a more active approach based on passive acoustic monitoring of the UNDEX events. There are different passive acoustic monitoring technologies and systems designed to characterize anthropogenic sound, and all of them can be used to automatically detect UNDEX events. Detection algorithms can be used in single-sensor monitoring devices, such as the one presented in [9], to alert of the presence of an UNDEX by means of surface buoys and surface telemetry. However, with the use of multiple vector sensors, UNDEX localization can be done even at distances up to sixteen thousand kilometers [10]. Recently, some authors have employed a compact array of acoustic vector sensors (1.25 m × 1.25 m) to locate sounds around 1 kHz [11]. Compact arrays of sensors provide a system that is easy to deploy, which in many situations gives sufficient localization accuracy. Surveillance vehicles such as remotely operated vehicles (ROVs) [12] or gliders [13] can also integrate UNDEX detection algorithms to perform mobile acoustic measurements in the immediate vicinity of a surveillance area.

The use of signal processing algorithms for the detection of UNDEX events might seem to be an easy task due to the high sound pressure levels that these events may reach. However, there are some practical problems that make simple detection algorithms cause problems and produce a large number of false positives [14]. There are some desirable characteristics that a robust UNDEX detector should have:

1. Pressure-level independent detection. This is necessary to be able to deal with the large dynamic range of these acoustic events. Possible situations may range from clipping in high intensity or very close-range UNDEX to low signal-to-noise ratios in the case of low intensity or detonations happening far away from the acoustic recorder.
2. Robust detection in the presence of non-UNDEX close-range sounds: The detector should be able to distinguish real UNDEX from other similar-duration transients or low-intensity sounds happening close to the acoustic recorder (non-UNDEX). In many cases, these acoustic events are close-range sounds such as nearby alpheid shrimps. In addition, signals with significant energy are generated when the hydrophone is physically impacted, which could be caused by grazing fish, for example [14].

Traditional machine-learning techniques for UNDEX detection found in the literature are based on obtaining the energy, duration [10,15], and some frequency-related parameters in order to obtain an acoustic signature [7]. Although these techniques provide fairly good detection percentages, they fail to detect UNDEX events in the two situations previously described. In this work we try to address the problem from a different perspective. We explore the arrow of time, a concept first introduced by Sir Arthur Eddington [16], to assess the feasibility of using arrow of time metrics in the characterization of UNDEX events. It must be noted that UNDEX are highly asymmetrical physical processes and therefore the thermodynamic arrow of time is high [17]. Even though the arrow of time has given good results in the characterization of economic series and the determination of the the playback direction of videos [18], and also in the characterization of electrocardiograms (ECG) [19], it has never been applied in the characterization of underwater acoustic events.

The rest of this work is structured as follows. In Section 2, we summarize the physics behind UNDEX and explain why the arrow of time metrics might help in the creation of robust UNDEX detectors. Later, in Section 3, we formally introduce the arrow of time as well as the different techniques that can be used when assessing it. We also give details on how these techniques can be employed in passive acoustic monitoring in general and in UNDEX events specifically. Then, in Section 4, we test the behavior of the different arrow of time metrics using simulated signals. We test them for different signal-to-noise ratios in the presence of clipping. In Section 5, we present a real application to assess the arrow of time metric in low-intensity UNDEX. We conclude the work discussing the possibilities and limitations of the proposed approach for the automatic detection of UNDEX.

## 2. General Description of an UNDEX

**Figure 1:**
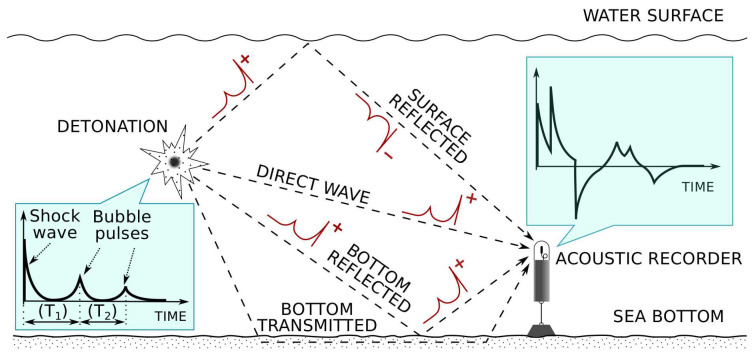
Main wave reflections produced by an UNDEX event received by a passive acoustic recorder moored at the bottom of the sea under constant speed of sound assumption.

The underwater detonation of an explosive charge can best be described as an exothermic chemical reaction that is self-sustaining after initiation [20]. It is a complex phenomenon, involving many aspects that need to be addressed in order to fully understand the development and propagation of the acoustic pressure wave through the fluid. In a simple approach, we can decompose the pressure wave into a primary shock wave due to the detonation itself followed by a series of bubble pulses (see the bottom left rectangular inset of Figure 1). The shock pulse has a rapid rise time and exponential decay, whereas the bubble pulses have an oscillating behavior due to expansion and contraction during the vertical migration of the highly pressurized gas bubble. Some low explosives, such as black powder, do not generate an instantaneous pressure rise or shock wave [21]. However, as a result of the gas bubble, successive bubble pulses and oscillations appear in both low-detonation and high-detonation explosives.

Some parameters of this oscillation convey information about the explosive yield. As an example, if the explosion is far from both the water surface and the bottom, (1) approximates the first bubble oscillation period ($T_{1}$) in seconds [22,23]),

(1) $$T_{1}=K\frac{W^{1/3}}{{\left(Z+10\right)}^{5/6}}$$

where *W* is the explosive yield in kilograms, *Z* is its depth in meters, and *K* = 2.11 for TNT.

All pressure waves generated at the explosive source propagate through the media to the observation point where the acoustic recorder is placed. The main contributions that need to be taken into account depend on the bathymetry, sound speed profile, and seabed geoacoustics. Some of the multipath contributions to be taken into account are the direct wave, the surface-reflected wave, the bottom reflected wave, and the bottom transmitted wave (the wave transmitted through the bottom materials and transmitted back into the water). Figure 1 represents a simplistic modeling approach for the process under constant speed of sound through the water column.

The smooth sea surface is a strong reflector of acoustic energy at nearly all frequencies. The reflection coefficient is close to −1, indicating that a 180-degree phase reversal occurs upon reflection.

The sea bottom, or seafloor, is a reflector of acoustic energy. However, in principle, the impedance contrast between water and bottom materials is smaller than in the water/air interface, although this depends on the type of sediment/bottom considered. Consequently, a large fraction of acoustic energy that impacts the seafloor will be transmitted into the bottom where it may reflect from sub-bottom layers.

When the charge is detonated very close to the sea surface, the reverse-polarity surface reflected signals can cancel out the direct path signal. In this scenario, the two main propagation paths are nearly identical in length, and, consequently, the two signals can arrive nearly simultaneously. Similarly, if the charge is detonated close to the sea floor, the two signals may partially overlap, causing constructive interference. The received signal strength is therefore a function of angle, frequency, and source depth.

As a result, an UNDEX produces a high pressure disturbance that propagates, producing a highly complex and disordered motion of water. As the disturbance moves away from the point of explosion the rate of entropy increase becomes slower [24]. The main hypothesis of this work is that (although weakly) the pressure waves reaching the underwater acoustic recorder might convey part of its complexity. Following the works by [24,25], we suggest that the arrow of time is an indirect method of measuring the complexity. As a result, we can use arrow of time measures as an indicator for the presence of UNDEX. It is important to highlight that UNDEX are not the only complex non-linear and dissipative events that have a strong arrow of time. For instance, seismic and underwater volcanic activity also have a strong arrow of time and thus arrow-of-time metrics should not be used as a standalone UNDEX detector.

## 3. Arrow of Time: Definition and Application Methods

The arrow of time is the “one-way direction” or “asymmetry” of time. The dynamics of many natural phenomena are traditionally modeled as if they had time reversibility, meaning that the statistical properties are identical when examined forwards and backwards in time [26]. However, this time symmetry is not always true and, in the real world, there are many examples of irreversible processes; e.g., financial time series, chaotic dissipative processes, nonlinear stochastic processes, and processes with memory operating away from thermodynamic equilibrium [27].

Many authors have studied the idea of time reversibility and have addressed its applicability to temporal series from different perspectives. Some of the most representative are the following: extracting a given feature that is related to the time-reversal asymmetry (e.g., the skewness of the slope distribution) and testing it against surrogates [28]; using a linear [29] or a non-linear predictor [26] to evaluate the statistical properties of the prediction error when examined both forwards and backwards in time; or performing a time series symbolization and subsequently analyzing the symbolized series using compression algorithms both forwards and backwards in time [30]. Recently, deep learning techniques have been proposed to capture the arrow of time in a Markov (decision) process [31].

Some of these approaches are not very appropriate when measuring the arrow of time in UNDEX events. For example, typical symbolization is local and requires some a priori knowledge about the timescale to be used in the temporal series. This information might not generally be available for UNDEX events because it depends on many unknown variables (explosive yield, distance of the detonation to the detector, propagation channel, etc.). Some other techniques such as testing against surrogates or deep learning techniques may be computationally intensive and may not be useful for real-time detectors. Additionally, it is important to stress that underwater acoustic events are usually non-stationary, which is a drawback when trying to evaluate the arrow of time. In fact, according to the original definition, non-stationary time series are infinitely irreversible, so the quantification of how irreversible a non-stationary time series is is an ill-defined problem that has not yet been satisfactorily solved [27]. Here, we will circumvent this problem with a careful selection of techniques that have proven to be able to quantify different degrees of irreversibility in both stationary and non-stationary processes.

Let x(n), 0<n<N−1 be a length *N* sequence obtained by sampling an underwater acoustic event with a sampling frequency $f_{s}=1/T_{s}$. Different techniques for assessing the arrow of time of the acoustic event x(n) are presented below.

### 3.1. Time-Reversal Asymmetry of Acoustic Events

A simple statistic to assess the arrow of time is the time-reversibility metric ($T_{REV}$), which is the mean of the slopes taken to the third power normalized by the standard deviation to the third power (${\hat{\sigma }}^{3}$) [32]. This metric is computed for a fragment of $N_{0}$ samples ($N_{0}<N$) as described in (2),

(2) $$T_{REV}=\frac{1}{{\hat{\sigma }}^{3}\cdot \left(N_{0}-\tau \right)}\sum_{n=\tau }^{N_{0}-1}{\left(\frac{x(n)-x(n-\tau )}{\tau \cdot T_{s}}\right)}^{3},$$

where τ is an integer value that is empirically determined to make the slope estimation less sensitive to noise. It is important to highlight that in the presence of clipping, which is something that can happen in UNDEX to a greater or lesser extent, the slope distribution is altered with a larger number of zero-slope intervals (the clipped regions). In order to make $T_{REV}$ less sensitive to clipping, the selection of the signal fragment $N_{0}$ is crucial. In the case of UNDEX events, the early shock waves arriving at the recorder are the primary shock waves resulting from the detonation itself; therefore, $N_{0}$ should be set to this region (of the order of $T_{1}$ from (1)). However, as we have stated, some of these samples may be clipped and, as a result, a sliding window of length $N_{0}$ is proposed to obtain the mean $T_{REV}$ in the first instants of the registered UNDEX signal.

The interpretation of the metric can be performed by taking into account that we estimate the skewness of the slope distribution. Assuming a unimodal distribution of the slopes, which is common in acoustic recordings, a negative time-reversal indicates that the negative slope is more frequent than the positive one. The inverse happens for a positive time-reversal. A zero value means that positive and negative slopes are balanced; therefore, no evidence of an arrow of time exists. Although this is true for symmetric distributions, it may not be true for asymmetric ones. We will use the absolute value $|T_{REV}|$ to measure the arrow of time.

### 3.2. Prediction Residuals of Acoustic Events

A different approach for the arrow-of-time assessment consists of examining the prediction error when the signal is predicted both forwards x(n)={x(0),⋯,x(N−1)} and backwards $x_{i}\left(n\right)=\left\{x\left(N-1\right),\cdots ,x\left(0\right)\right\}$ in time. We used an autoregressive integrated moving average (ARIMA) model as well as a normalized nonlinear gradient descent (NNGD) predictor to analyze the UNDEX time series. Estimation errors were obtained: e(n) for the estimation error of the forward time series x(n); and $e_{i}\left(n\right)$ for the estimation error of the backwards time series $x_{i}\left(n\right)$.

**Figure 2:**
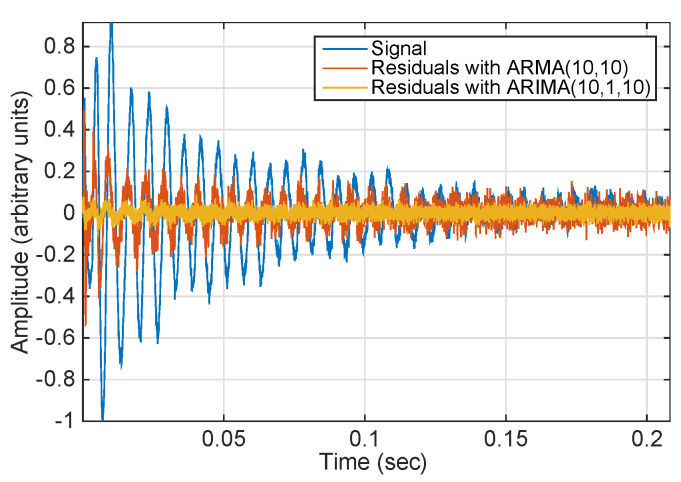
Example of how ARIMA(10,1,10) achieves better modeling (smaller residuals) than ARMA(10,10) in signals having a trend similar to that of an UNDEX event.

- The ARIMA model: Although some authors propose using a causal autoregressive moving average model (ARMA) [29,33] due to non-stationary behavior, we propose using an ARIMA(p,d,q) model for acoustic events. ARIMA models follow signals that have the stronger trends that UNDEX events have much better than ARMA models. The three components (p,d,q) are, respectively, the AR order, the degree of differencing, and the MA order of the model. If the three components are not properly chosen the model does not fit and the residuals will have a trend. Figure 2 illustrates this.
- The NNGD predictor: The NNGD algorithm [34] has been proven to outperform other adaptive algorithms when predicting nonlinear and non-stationary signals and has been used to forecast one sample ahead into the future of the acoustic events. The prediction error was obtained both forwards and backwards in time.

An analysis of the error when using both linear and non-linear predictors was performed by means of the following metrics:

(i) The predictive residuals sign-test metric ($PR_{ST}$): This metric shows the relative frequency for which the absolute magnitude of each forward prediction error e(n) exceeds the reverse prediction error $e_{i}\left(n\right)$.
  (3) $$PR_{ST}=P\left(|e\left(n\right)|>|e_{i}\left(n\right)|\right)$$
  P(·) estimates the probability. If the predictions are equally accurate when the time series is examined in the forward direction as when analyzed under time reversal, the value $PR_{ST}=0.5$. Values of $PR_{ST}$ that are larger or smaller than 0.5 indicate time directionality to some extent. We use $|PR_{ST}-0.5|$ as the arrow-of-time metric. The higher the metric the more evident the arrow of time is in the acoustic event.
(ii) The predictive correlation coefficient ($PR_{CC}$): This is the Pearson correlation coefficient, $r_{\hat{x}x}$, between real forward values x(n) and predicted forward values, $\hat{x}\left(n\right)=x\left(n\right)-e\left(n\right)$, as compared with the correlation coefficient, $r_{{\hat{x}}_{i}x_{i}}$, between real backward values $x_{i}\left(n\right)$ and predicted backward values, ${\hat{x}}_{i}\left(n\right)=x_{i}\left(n\right)-e_{i}\left(n\right)$. The metric is defined by:
  (4) $$PR_{CC}=\frac{r_{\hat{x}x}}{r_{{\hat{x}}_{i}x_{i}}}.$$
  The ratio $PR_{CC}$ should be close to 1 when no arrow of time exists. We then use $|PR_{CC}-1|$ as the arrow-of-time metric. As before, the higher the metric, the more evident the arrow of time will be.

The combination of the two predictors (ARIMA and NNGD) with the two metrics produces four different ways of assessing the arrow of time: $PR_{ST}^{ARIMA}$, $PR_{CC}^{ARIMA}$, $PR_{ST}^{NNGD}$, and $PR_{CC}^{NNGD}$.

### 3.3. Visibility Graphs of Acoustic Events

**Figure 3:**
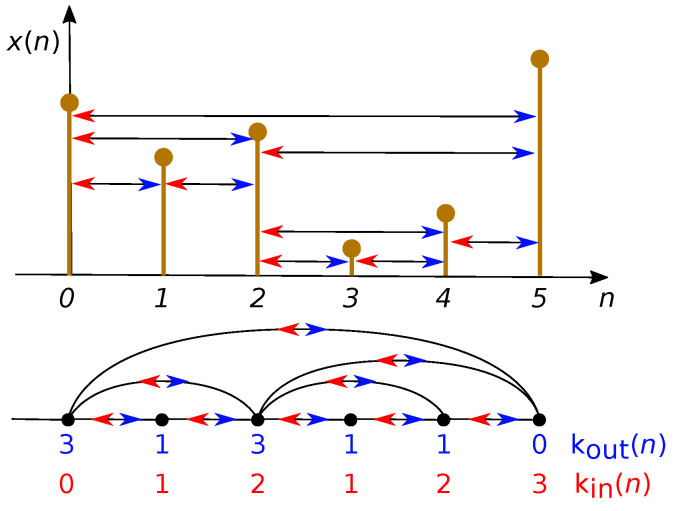
Illustration of the way the horizontal visibility graph is obtained for a time series x(n) according to the criteria in (5).

Visibility algorithms are a collection of methods that map series to networks according to specific geometric criteria [35]. In this way, some powerful graph theory tools are used to provide alternative time series characterization. One of these methods, which is used in the present work, is the so-called direct horizontal visibility graph (DHVg). We can create the horizontal visibility graph of x(n) by checking if two samples, *i* and *j*, are horizontally connected. The criterion to be satisfied is that the two samples, *i* and *j*, are connected if one can draw a horizontal line joining the two samples that does not intersect any intermediate sample. The geometrical condition is described in (5) and the idea is illustrated in Figure 3.

(5) x(i),x(j)>x(n),∀n∣i<n<j.

As a result, each sample *n* has an out-going degree $k_{out}^{x}\left(n\right)$ that can be obtained by counting all of the samples to which sample *n* is horizontally connected (blue arrows in Figure 3). The out-going degree $k_{out}^{x}\left(n\right)$ is related to the number of links with future nodes. Similarly, an in-going degree $k_{in}^{x}\left(n\right)$ can be defined that is related to the number of links with past nodes (red arrows in Figure 3). The information stored in the $k_{in}^{x}\left(n\right)$ and $k_{out}^{x}\left(n\right)$ distribution takes into account the amount of time irreversibility of the associated series. As a first approximation, this can be measured as the distance (in the distribution sense) between the probability density function of $k_{in}^{x}\left(n\right)$, named $P_{in}^{x}\left(k\right)$, and that of $k_{out}^{x}\left(n\right)$, named $P_{out}^{x}\left(k\right)$.

In order to enhance the overall results of the DHVg technique when applied to passive acoustic monitoring and detection of UNDEX events, we propose working with the instantaneous phase ψ(n) estimated by means of the Hilbert transform. The water shock contributions and reflections present in UNDEX events introduce phase shifts in the received signals x(n), so the instantaneous phase is well suited for the characterization of these shock waves through the DHVg. Some researchers have already demonstrated the importance of the phase for noise robust characterization of acoustic events in both aerial [36] and underwater sound events [37]. Thus, (5) was rewritten as:

(6) ψ(i),ψ(j)>ψ(n),∀n∣i<n<j,

where $\psi \left(t\right)=atan\frac{ℑ(H[x(n)])}{x(n)}$ and ℑ(·) is the imaginary part, and H[·] indicates the Hilbert transform. Visibility graph analysis of the instantaneous phase provides the probability functions $P_{in}^{\psi }\left(k\right)$ and $P_{out}^{\psi }\left(k\right)$. The distance between the *in* and *out* degree distributions can be measured by making use of the Kullback–Leiber divergence [35]. We define the VGs Kullback–Leiber Divergence metric ($VG_{KLD}$) as:

(7) $$VG_{KLD}=D\left[P_{out}^{\psi }\left(k\right),P_{in}^{\psi }\left(k\right)\right]=\sum_{k}P_{out}^{\psi }\left(k\right)\cdot log\frac{P_{out}^{\psi }\left(k\right)}{P_{in}^{\psi }\left(k\right)},$$

where log is the logarithmic function. When no arrow of time exists, both distributions should be similar and the distance close to 0. Higher distances indicate the presence of an arrow of time.

## 4. Montecarlo Simulations over Synthetic Signals

Simulations were carried out for a low explosive (like black powder), which does not have an instantaneous pressure rise. As a result, we modeled only the sound pressure wave obtained for a collapsing and rebounding spherical bubble using the Gilmore equation for an initial bubble depth equal to the depth of the UNDEX [38]. In order to provide some statistical variability to the simulations, a very simple ray-tracing model was used. Our current intent is not to perform a rigorous modeling of the propagation channel, but rather to that take into account the constructive/destructive interferences of the broadband acoustic waves as a result of the UNDEX. For that purpose we used three contributions [20]: direct wave, bottom reflected, and surface reflected (see Figure 1). Details of the ray-tracing simulation were as follows: seabed bottom at 50 m, receiver depth 40 m, source depth randomly distributed in the range 6–50 m, and distance from source to receiver 1.5 km.

For non-UNDEX acoustic events, these sounds have been modeled as a damped oscillation of random frequency ($f_{0}$) that is uniformly distributed in the frequency range where most of the energy of UNDEX events is concentrated (100–600 Hz). This particular modeling of the non-UNDEX events given by (8) was chosen empirically after examining close-range misclassified events. In the simulations, the values of A = 10 and σ=17 were used to mimic the attenuation in time that is present in UNDEX events.

(8) $$x\left(n\right)=A\cdot e^{-\sigma \cdot n/f_{s}}\cdot sin\left(2\cdot \pi \cdot f_{0}\cdot n/f_{s}\right),\;n\geq 0$$

**Figure 4:**
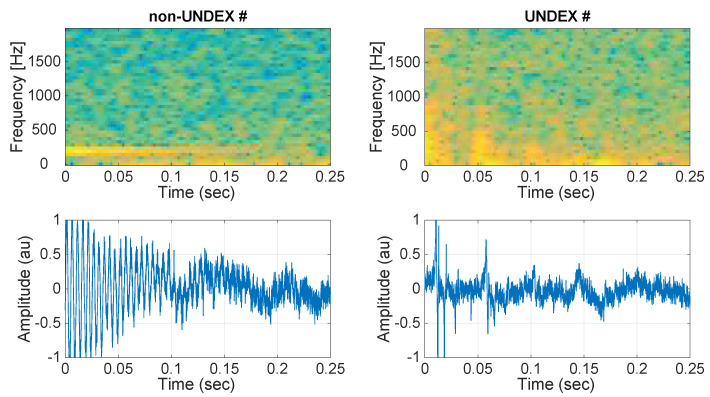
Example of simulated non-UNDEX and UNDEX events (although they are very different, distinguishing between them may be challenging when they are severely clipped).

The same ray-tracing model was used for the non-UNDEX events. Figure 4 illustrates the simulated UNDEX and non-UNDEX events. All of the arrow-of-time metrics described in the previous section were tested in the simulated events for different signal-to-noise ratios (SNRs), both with and without clipping.

Although events of 1 s duration at $f_{s}$ = 24,000 Hz were simulated, only a small region at the beginning of each event was used to evaluate the arrow of time with the proposed metrics. It is in this region where most of the energy of the event is located and there should be a stronger indicator of the time asymmetry. The specific details of how many milliseconds were employed for each metric as well as some other settings are given in Table 1.

**Figure 5:**
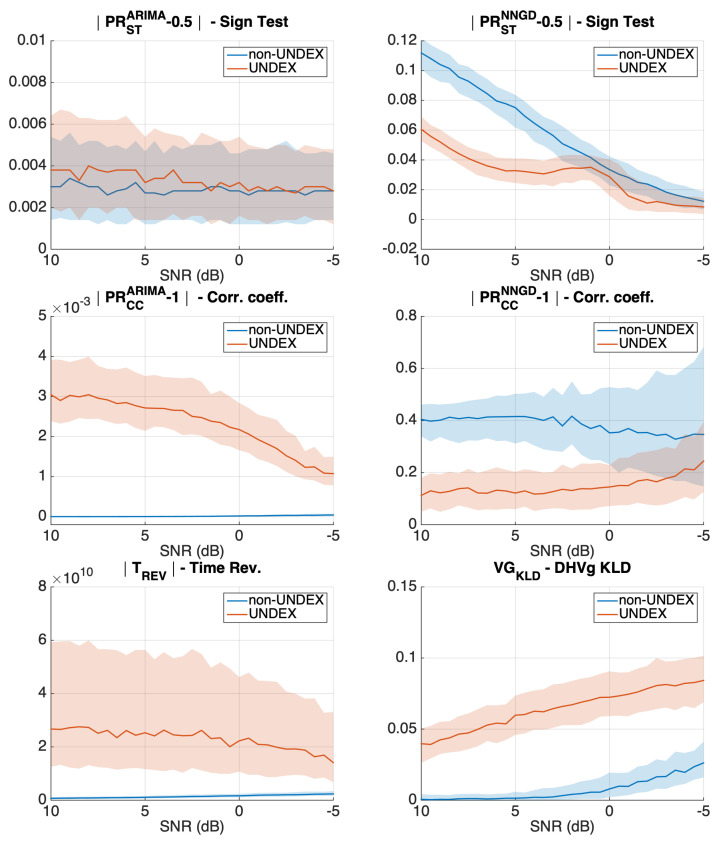
Evolution of all the presented metrics in simulated events when the SNR decreases (Y axis in arbitrary units). The results were obtained for 500 Monte Carlo runs and for unclipped acoustic events.

**Figure 6:**
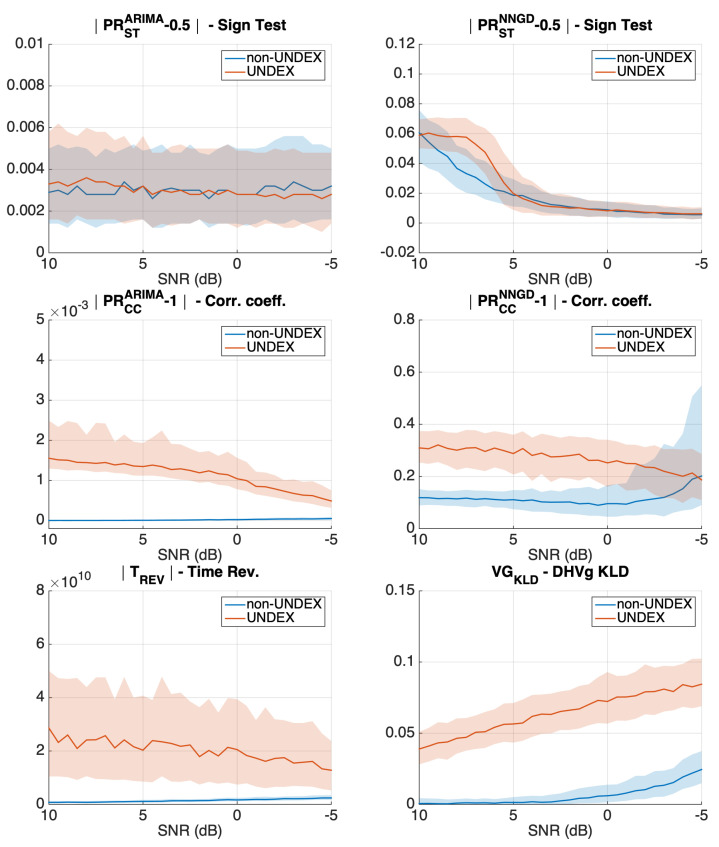
Evolution of all the presented metrics in simulated events when the SNR decreases (Y axis in arbitrary units). The results were obtained for 500 Monte Carlo runs for events clipped to 50% of their maximum value.

Figure 5 and Figure 6 show the results when adding different amounts of pink noise to obtain a decreasing SNR from 15 dB to −5 dB. In each of the panels and for each of the acoustic events (UNDEX and non-UNDEX), the continuous line represents the median of 500 Monte Carlo runs, whereas the shadow region represents the interquartile range (the difference between the 25th and the 75th percentiles). Vertical axis limits were identically set for the same metric between clipped and unclipped simulations.

The simulation results show that the sign-test for the ARIMA model (top left panel of Figure 5) is not able to clearly distinguish between UNDEX and non-UNDEX events. However, the correlation coefficient metric was successful at the task (middle left panel). In the case of the NNGD model, the linear non-UNDEX events were not accurately predicted by the non-linear predictor. This makes both the sign-test and the correlation coefficient have inconsistent metrics with a higher value for the UNDEX events than that obtained for the non-UNDEX events (top and middle right panels). The time reversal (bottom left panel) and visibility graph metrics (bottom right panel) also showed some potential for assessing the arrow of time in simulated events, even in low SNR.

When clipping is added for the ARIMA model (Figure 6), the arrow-of-time identification by means of the correlation coefficient metric diminishes due to the fact that linear models cannot accurately predict the clipped acoustic events. In the case of the NNGD, since both UNDEX and non-UNDEX events become nonlinear, the NNGD achieves a better modeling and the obtained sign-test and correlation coefficient metrics agree with what was expected (higher metric values for the UNDEX events than those obtained for non-UNDEX). However, only the correlation showed some potential for distinguishing between the two acoustic events in low SNR. Time reversibility is also affected by the clipping, but it still maintains some capacity to measure the arrow of time. The visibility graph metric (KLD distance of the DHVg) is the metric that was the least sensitive to clipping in simulated signals.

## 5. Application to Real UNDEX Events

A real situation where UNDEX of low intensity can occur is in the process of tuna harvesting, especially blue fin tuna (BFT). Some BFT farms use a spear gun with a shotgun shell at the end of a long stick (2–3 m) that is propelled from a spear gun with elastic/rubber bands, detonating the shotgun shell upon contact [39]. This guarantees that death occurs as swiftly as possible and prevents the formation and build-up of lactic acid, which decreases the quality of the meat.

It is important to highlight that a shotgun shell has an approximate load of 40–45 grains (2.59–2.9 g). TNT equivalent can be obtained using the relative effectiveness factor (RE factor) of black powder, which is 0.55. The TNT has an RE of 1, so the 2.6–2.9 g are equivalent to 1.4–1.6 g of TNT. Thus, the load of one of these detonations is clearly under the limit that an underwater detonation must have in order to be included in the EU MSFD impulsive noise register in the category of very low (8 g to 210 g).

**Figure 7:**
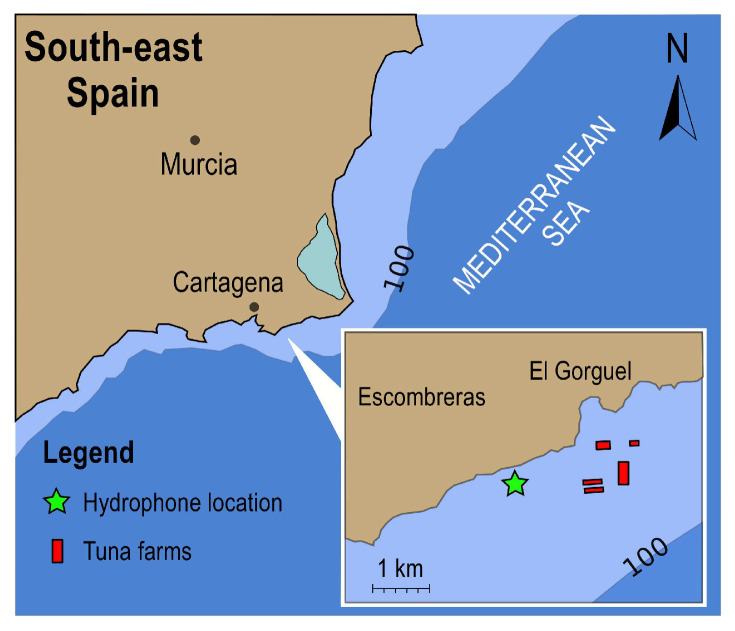
Cartagena region, showing the tuna farm and the UNDEX sites (red squares) as well as the hydrophone location (green star). The distance from the UNDEX to the hydrophone was approximately 1.2 km.

An acoustic campaign was performed in the Mediterranean sea in the area of Cartagena (Spain). Recordings were performed with a SAMARUC passive acoustic monitoring device (Universitat Poliècnica de València) [40] with a Cetacean Research hydrophone (C57) and a sampling frequency of 48 kHz. The recorder was deployed at a depth of 50 m, close to a tuna farm where the harvesting was performed using the lupara shotgun technique (see Figure 7).

**Figure 8:**
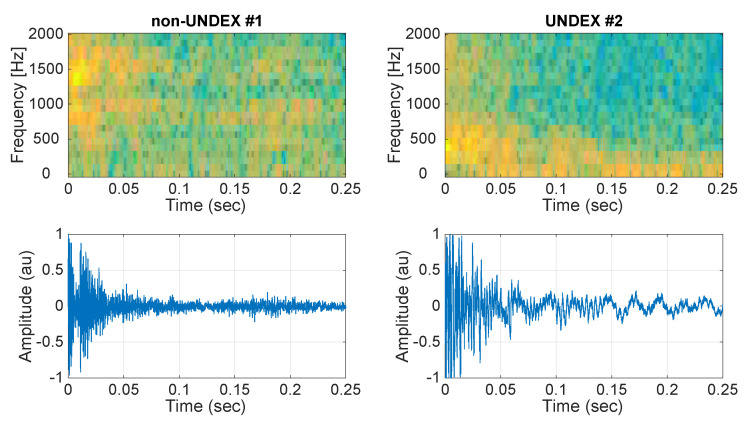
Example of a non-UNDEX event and a real UNDEX event from the database over the duration of the first 0.25 s.

The recordings started in October 2018 and lasted for a month with a duty cycle of 5 min on/10 min off. Preliminary detection of the acoustic events for the impulsive database creation was performed with a short time average/long time average (STA/LTA) detector, which is a well-known seismic impulsive event detector [41]. Settings for the STA/LTA detector were as follows: the short time-window duration was 1 s; the long time-window duration was 8 s; and the mean power in the short time-window was 10 times larger than that in the long time-window. The output of the STA/LTA detector gave 132 events with a duration of 1 s. An inspection of the database by an expert acoustic technician showed that the database was composed of UNDEX events as well as other impulsive events that, in many cases, resemble UNDEX events. The events were carefully analyzed and classified as UNDEX, non-UNDEX, and UNSURE categories. Many of the events had some degree of clipping, which made the classification of the events difficult. Figure 8 shows an example of two real non-UNDEX and UNDEX events from the recording campaign.

**Figure 9:**
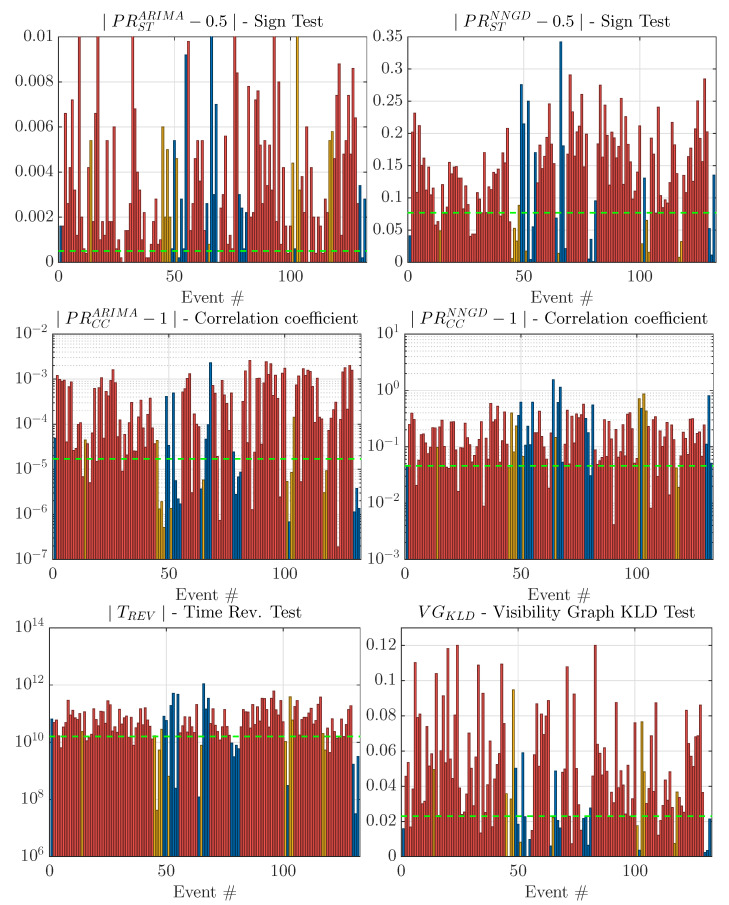
Assessment of the arrow-of-time metrics in the database of real events (Y axis in arbitrary units). The colors indicate the human expert classification: red corresponds to UNDEX, blue corresponds to non-UNDEX, and yellow corresponds to UNSURE categories. The threshold (dashed green line) was set to obtain a fixed detection of UNDEX events equal to 90%.

The 132 potential acoustic events were also evaluated with the arrow-of-time metrics proposed in the previous sections. The results are presented in Figure 9. The figure also shows a threshold level (dashed green line) that was obtained so that the detection probability of UNDEX events remains equal to 90% in all of the metrics. With this threshold, we obtained the probability of correct classification (PCC) and the probability of false classification (PFC) of non-UNDEX events. The results are shown in Table 2 where the UNSURE events (yellow bars) were not taken into account for the statistics.

The analysis of Table 2 shows that the metrics $PR_{ST}^{ARIMA}$ and $PR_{CC}^{NNGD}$ were not able to show any clear arrow-of-time indication. The metrics $PR_{CC}^{ARIMA}$, $PR_{ST}^{NNGD}$, and $T_{REV}$ gave a detection probability that was slightly superior to 50%. Although this indicates the presence of a higher arrow of time in UNDEX events compared to non-UNDEX events, this is not enough if these metrics are used in the design of an automatic detector. The metric obtained from the DHVg of the instantaneous phase, the $VG_{KLD}$ metric, was the only one that had a PCC/PFC in a range that might be considered acceptable for automatic classification of UNDEX/non-UNDEX events. This behavior agrees quite well with what was shown in the simulations, where the $VG_{KLD}$ was the metric that was the least influenced by the presence of clipping.

## 6. Conclusions

In this paper, we have presented a rigorous study of the different techniques for assessing the arrow of time and their application to underwater acoustic events in general and to UNDEX events specifically. We have shown through simulations and real signal analysis that the pressure waves resulting from UNDEX carry some features (complexity) that indicate the nonlinear and dissipative process originating from them. These features can be assessed using different arrow-of-time metrics and can be used for the characterization of UNDEX events as well as for distinguishing them from similar impulsive non-UNDEX events. Furthermore, in this paper, we have proposed a new metric that is based on evaluation by means of the direct horizontal visibility graphs of the instantaneous phase. This new metric obtained good results, surpassing all other arrow-of-time metrics that are traditionally used, even when dealing with clipped events. Overall, the arrow-of-time assessment of real underwater acoustic events has produced promising results and provided a classification that was very similar to that obtained by an expert analyst. However, the detection percentages were not as high as the ones obtained with simulated signals. A feasible explanation for this might be based on the fact that a realistic channel model was not adopted and that evidence of the arrow of time diminishes in long-range detonations due to frequency-selective attenuation and noise. Therefore, in order to develop surveillance systems and automatic UNDEX detectors, we consider that the arrow-of-time metric proposed here should be combined with machine learning and time-frequency features. Future research on this topic that should be addressed includes the following: studying how to combine arrow-of-time metrics with traditional ones, performing more real UNDEX measures at different ranges and with different explosive types; and performing simulations with realistic broadband acoustic propagation models (both in shallow waters and deep water channels). Even though the use of arrow-of-time metrics might be limited to UNDEX that occur close to the receiver, some of the obtained results could be be valuable in designing detectors for situations when a large amount of energy is released, such as tsunami waves from submarine earthquakes.

## Figures and Tables

**Table 1:** Setting details used for the different arrow-of-time metrics.

Arrow-of-Time Metric	Setting Details
Slope time-reversal	N = 5000 (208 msec.),
	$N_{0}=2400$ (100 msec.),
	τ=5.
Prediction residuals (ARIMA)	N = 5000 (208 ms.),
	ARIMA (12,1,12)
Prediction residuals (LMS)	N = 5000 (208 ms.),
	tap size: 10 samples [34]
Visibility graphs	N = 1000 (41 msec.)

**Table 2:** Probability of correct and false classifications for a fixed constant UNDEX detection of 90%. Results obtained when applying the metrics to the database containing UNDEX and non-UNDEX ($\bar{UNDEX}$) events.

	${\mathrm{PFC}}_{\mathrm{UNDEX}}$	${\mathrm{PCC}}_{\mathrm{UNDEX}}$	${\mathrm{PFC}}_{\bar{\mathrm{UNDEX}}}$	${\mathrm{PCC}}_{\bar{\mathrm{UNDEX}}}$
$PR_{ST}^{ARIMA}$	10%	90%	89%	11%
$PR_{CC}^{ARIMA}$			42%	57%
$PR_{ST}^{NNGD}$			47%	53%
$PR_{CC}^{NNGD}$			89%	11%
$T_{REV}$			47%	53%
$VG_{KLD}$			21%	79%

## Data Availability

The data presented in this study are available on request from the corresponding author and on his personal website.
